# Role of *TLR4*  rs4986790A>G and rs4986791C>T Polymorphisms in the Risk of Inflammatory Bowel Disease

**DOI:** 10.1155/2015/141070

**Published:** 2015-05-18

**Authors:** Ran Ao, Ying Wang, Dao-Rong Zhnag, Ya-Qi Du

**Affiliations:** ^1^Department of Gastroenterology, First Affiliated Hospital of China Medical University, Shenyang 110001, China; ^2^Department of Pathophysiology, College of Basic Medical Sciences, China Medical University, Shenyang 110001, China

## Abstract

*Objective*. The present meta-analysis investigated the contribution of* TLR4* rs4986790A>G and rs4986791C>T genetic polymorphisms in increasing the risk of inflammatory bowel disease (IBD).* Methods*. Databases were searched using a combination of keywords related to* TLR4* and IBD. Relevant studies were selected based on strict inclusion and exclusion criteria. Meta-analysis of the data extracted from the selected studies was performed using CMA 2.0 statistical analysis software.* Results*. Out of the 70 studies retrieved by database search, only 13 studies were eligible for inclusion in this meta-analysis and these 13 studies contained a total number of 4409 IBD patients and 5693 healthy controls. The meta-analysis results demonstrated that* TLR4* rs4986790A>G polymorphism is associated with an increased risk of IBD (allele model: OR = 1.268, 95% CI = 1.124~1.431, and *P* < 0.001; dominant model: OR = 1.240, 95% CI = 1.090~1.409, and *P* = 0.001). Similarly, TLR4 rs4986791C>T polymorphism also conferred an increased risk of IBD (allele model: OR = 1.259, 95% CI = 1.092~1.453, and *P* = 0.002; dominant model: OR = 1.246, 95% CI = 1.072~1.447, and *P* = 0.004).* Conclusion*. Our meta-analysis results demonstrate that* TLR4* rs4986790A>G and rs4986791C>T genetic polymorphisms are associated with the etiopathogenesis of IBD.

## 1. Introduction

Inflammatory bowel disease (IBD) mainly consists of two types, Crohn's disease (CD) and ulcerative colitis (UC), both of which are chronic inflammatory disorders of unknown etiology [1]. Northern Europe and North America show high incidence rates of pediatric IBD [2]. In particular, UC and CD together affect approximately 1 in 250 among European, North American, and Australasian populations [3]. UC is characterized by inflammation of the colonic mucosa that spreads proximally from the anus, while, in CD, the inflammatory lesions may occur in any area of the gastrointestinal (GI) tract and involve the entire thickness of the intestinal wall, exhibiting focal characteristics [4, 5]. The etiopathogenesis of IBD is unknown, but genetic predisposition, gut microbial flora, and environmental factors are suspected to play important roles. For example, abnormal immune response by the host towards gut microbes is implicated in the pathogenesis of IBD. Similarly, environmental triggers, including nonsteroidal anti-inflammatory drugs, antibiotics, and bacterial and viral infections, are risk factors that enhance the severity of IBD [5–7]. Previous studies suggest a significant contribution of genetic factors to the etiology of IBD and an increased IBD risk is observed in individuals with a family history of IBD [2]. With recent advances in complementary technologies to uncover genetic risk factors for human diseases, more than 40 IBD susceptibility loci have been identified through linkage analysis, association mapping, and candidate gene association studies [5, 8]. Most importantly, imbalances in pro- and anti-inflammatory cytokine levels, resulting from the altered expression of genes involved in innate immunity, are implicated in the development and unremitted course of IBD [9].

Toll-like receptor 4 (TLR4) is important for host immune response to pathogens and has a specific role in inflammatory pathways [10]. TLR4 is also implicated in colitis-associated neoplasia and thus is intimately linked to IBD onset and progression. TLR4 (EMBL/GenBank/DDBJ accession number U93091) maps to chromosome 9q32-33 and is a member of an emerging family of molecules implicated in innate immune recognition [11]. TLR4 is the main receptor for lipopolysaccharide (LPS) expressed in macrophages, dendritic cells, endothelial cells, and the intestinal epithelium [12]. TLR4 mediates lipopolysaccharide-induced maturation and activation of myeloid dendritic cells [10]. Importantly, TLR4 signaling in immune cells of the colonic mucosa plays a central role in maintaining chronic inflammatory state by producing inflammatory cytokines [13]. Recent results from animal models show that tissue inflammation correlating with IL-17 production is mediated by TLR4 signaling in chronic inflammatory diseases [14]. Additionally, an imbalance in T cell subsets, such as T regulatory cells, Th1, Th2, and Th17, and the resulting altered profile of cytokine secretion from these cells, influences the susceptibility to IBD [15]. Consistent with this, an upregulation of TLR4 in the colonic mucosal epithelium and in myeloid dendritic cells is reported in active IBD [16]. Two single nucleotide polymorphisms (SNPs) in the* TLR4 *gene, D299G (299 A/G, Asp299Gly, and rs4986790A>G) and T399I (399 C/T, Thr399Ile, and rs4986791C>T), appear to be prominent in host interactions with the environment [15]. These functional* TLR4* polymorphisms modulate innate host defense responses against infections and their frequency in various populations is thought to be influenced by selection pressures, which is dependent on the interactions with local pathogens [17]. A study carried out in a Caucasian population reported that* TLR4 *rs4986790A>G and rs4986791C>T SNPs conferred a high risk of developing IBD [12]. However, a study conducted in a Han population contradicted these findings and showed no significant association between* TLR4* rs4986790A>G polymorphism and IBD susceptibility [18]. Therefore, the present meta-analysis was conducted to examine the association between the two* TLR4* polymorphisms, rs4986790A>G and rs4986791C>T, and IBD susceptibility.

## 2. Methods and Materials

### 2.1. Literature Search

PubMed, EBSCO, Ovid, Springerlink, Wiley, Web of Science, Wanfang databases, China National Knowledge Infrastructure (CNKI), and VIP databases were searched to identify relevant studies published prior to October 2014. Manual search of cross-references was also conducted to identify additional studies. The search involved a combination of keywords and free words related to inflammatory bowel disease (IBD), ulcerative colitis (UC), Crohn's disease (CD), and TLR4 using a highly efficient and sensitive searching strategy: (“Inflammatory Bowel Diseases” or “Colitis, Ulcerative” or “Crohn Disease”) and (“Toll-Like Receptor 4” or “TLR-4” or “TLR 4”) and (“Polymorphism, Genetic” or “SNP”). Database search was restricted to studies published in Chinese and English languages.

### 2.2. Inclusion and Exclusion Criteria

The inclusion criteria for selection of studies for this meta-analysis were as follows: (1) studies should be related to IBD risk and* TLR4 *gene polymorphisms rs4986790A>G and rs4986791C>T; (2) study design should be case-control study; (3) subjects in the enrolled studies should be patients with confirmed IBD diagnosis and healthy subjects as controls; (4) outcome index is as follows: allele and genotype frequencies in case and control groups; (5) only studies published in Chinese and English languages were selected. The exclusion criteria were as follows: (1) summary and abstracts were not selected; (2) animals studies were excluded; (3) duplicate publications were not used; (4) studies that provided insufficient information were not used; (5) for overlapping publications, only the most recent or the most complete study was included in this meta-analysis.

### 2.3. Data Extraction and Quality Assessment

Data was extracted from each study by two independent investigators and the following information was collected: surname and initials of the first author, year of submission, country, ethnicity, language, age, gender, cases, detective methods, research designs, and single nucleotide polymorphism (SNP). Disagreement on the inclusion of any study was resolved by consultation with a third investigator.

### 2.4. Statistical Analysis

Comprehensive Meta-Analysis 2.0 software (Biostatic Inc., Englewood, New Jersey, USA) was used for statistical analysis of the extracted data. The differences in allele and genotype frequencies of* TLR4 *rs4986790A>G and rs4986791C>T polymorphisms were compared using odds ratio (OR) and its 95% confidence intervals (95% CI). *Z* test was carried out to evaluate the significance of overall effect values [19]. Forest plots were analyzed to compare ORs and 95% CI between case and control groups. The heterogeneity between the studies was evaluated with Cochran's Q-statistic (*P* < 0.05 was considered significant) and *I* ^2^ test (0%: no heterogeneity; 100%: maximal heterogeneity) [19, 20]. In order to calculate the pooled ORs, fixed or random effects models were used. When significant heterogeneity was observed (*P* < 0.05 or *I* ^2^ > 50%), a fixed effects model was used; otherwise, the random effects model was employed [21]. Univariate and multivariate metaregression analysis of outcomes were utilized to identify potential sources of heterogeneity and Monte Carlo simulation was employed for further confirmation [22–24]. Sensitivity analysis was performed to evaluate whether removal of any single study influenced the overall outcomes. The funnel plot, classic fail-safe *N*, and Egger's linear regression test were used to assess publication bias to ensure the reliability of the results [25–27]. A bilateral test was conducted, with *P* < 0.05 considered to be significant.

## 3. Results

### 3.1. Selection of Eligible Studies

**Figure 1:**
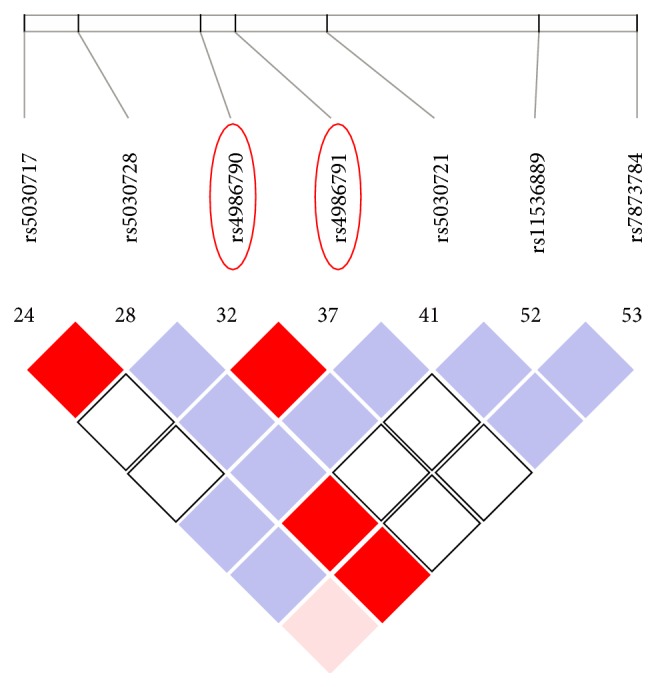
Schematic diagram of the position of* TLR4 *gene (rs4986790A>G and rs4986791C>T).

Electronic database and manual searches resulted in the identification of 70 potential articles. After excluding duplicates (*n* = 9), nonhuman studies (*n* = 2), letters, reviews, non-English or non-Chinese studies (*n* = 3), studies that were not case-control design (*n* = 13), and studies unrelated to our research topic (*n* = 22), a total of 17 studies remained for full-text review. Following the inclusion and exclusion criteria, 1 study without statistics and 3 studies with insufficient statistics were eliminated. Finally, 13 studies [10, 15, 16, 28–37] published between 2004 and 2013 were included in the current meta-analysis and contained 4409 IBD patients (1997 UC patients and 2412 CD patients) and 5693 healthy controls. Of these, 10 studies were conducted in Caucasian population and 3 studies were in Asian population. Two major* TLR4 *SNPs, rs4986790A>G and rs4986791C>T, were involved in the meta-analysis (Figure 1).  Table 1 shows the minimum minor allele frequency (MAF) of the SNPs in different ethnic groups from HapMap, which illustrates the pattern of MAFs in major ethnic groups. SNP detection methods in the 13 studies were polymerase chain reaction with the restriction fragment length polymorphism (PCR-RFLP), allele-specific polymerase chain reaction (AS-PCR), and TaqMan assay. Baseline characteristics of the 13 eligible studies are summarized in Table 2.

### 3.2. Meta-Analysis of the Association between* TLR4* Gene rs4986790A>G Polymorphism and IBD Risk

**Figure 2:**
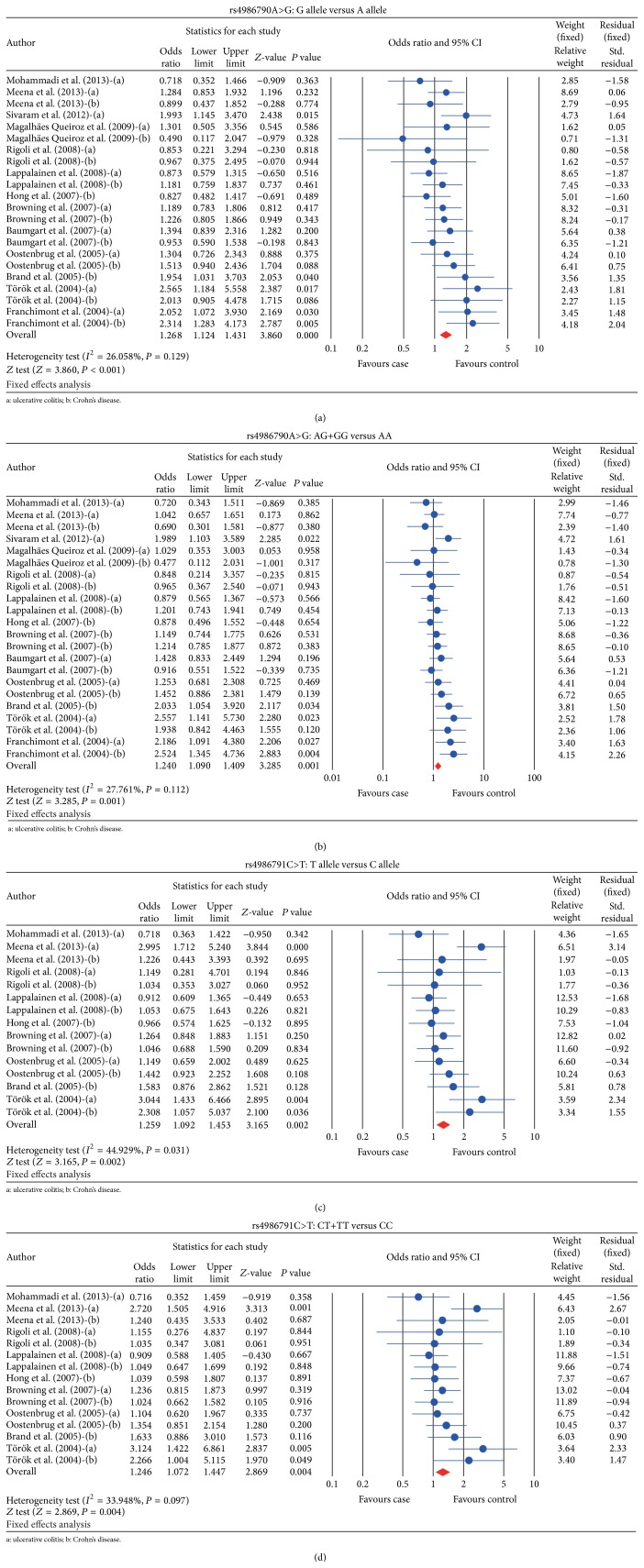
Forest analyses of the differences in allele and genotype frequencies of* TLR4 *rs4986790A>G and rs4986791C>T polymorphism between case and control groups. (a) Forest analyses of the differences in allele frequencies of rs4986790A>G. (b) Forest analyses of the differences in genotype frequencies of rs4986790A>G. (c) Forest analyses of the differences in allele frequencies of rs4986791C>T. (d) Forest analyses of the differences in genotype frequencies of rs4986791C>T.

**Figure 3:**
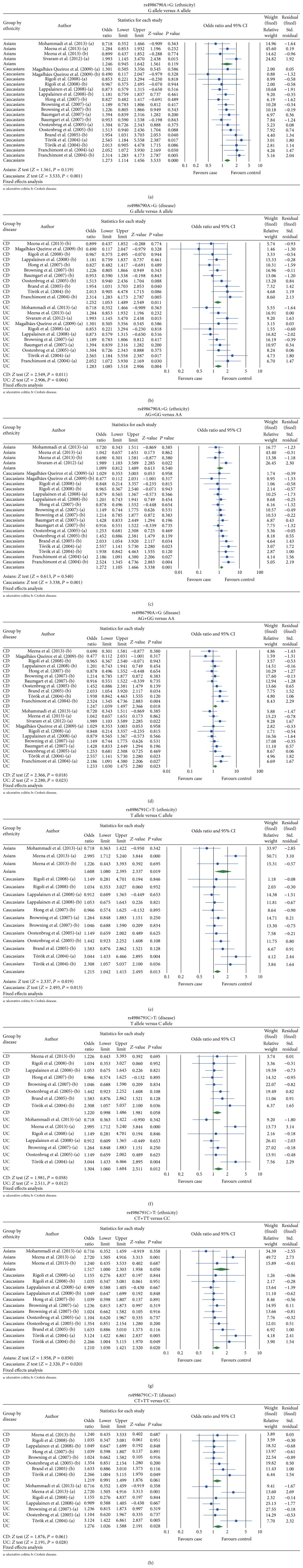
Subgroup analysis of the differences in allele and genotype frequencies of TLR4 rs4986790A>G and rs4986791C>T polymorphism between case and control groups. (a) Ethnicity analysis of the differences in allele frequencies of TLR4 rs4986790A>G; (b) disease analysis of the differences in allele frequencies of TLR4 rs4986790A>G; (c) ethnicity analysis of the differences in genotype frequencies of TLR4 rs4986790A>G; (d) disease analysis of the differences in genotype frequencies of TLR4 rs4986790A>G; (e) ethnicity analysis of the differences in allele frequencies of TLR4 rs4986791C>T; (f) disease analysis of the differences in allele frequencies of TLR4 rs4986791C>T; (g) ethnicity analysis of the differences in genotype frequencies of TLR4 rs4986791C>T; (h) disease analysis of the differences in genotype frequencies of TLR4 rs4986791C>T.

Thirteen studies investigated the association between* TLR4* gene rs4986790A>G polymorphism and IBD risk. No heterogeneity was observed in this meta-analysis (allele model: *I* ^2^ = 26.058%, *P*_*h*_ = 0.129; dominant gene model: *I* ^2^ = 27.761%, *P*_*h*_ = 0.112), and thus fixed effects model was adopted. Our findings demonstrated that* TLR4 *rs4986790A>G polymorphism significantly increased IBD risk (allele model: OR = 1.268, 95% CI = 1.124~1.431, and *P* < 0.001; dominant gene model: OR = 1.240, 95% CI = 1.090~1.409, and *P* = 0.001) (Figures 2(a) and 2(b) and Table 2). Subgroup analysis based on ethnicity demonstrated that rs4986790A>G increased the IBD risk in Caucasians (allele model: OR = 1.273, 95% CI = 1.114~1.456, and *P* < 0.001; dominant model: OR = 1.272, 95% CI = 1.105~1.466, and *P* = 0.001); but this relationship was not found in Asian population (allele model: OR = 1.246, 95% CI = 0.945~1.642, and *P* = 0.119; dominant model: OR = 1.099, 95% CI = 0.812~1.489, and *P* = 0.540) (Figures 3(a) and 3(b)). Subgroup analysis based on IBD types suggested that the* TLR4* rs4986790A>G polymorphism conferred an increased risk of both UC and CD (CD: allele model: OR = 1.242, 95% CI = 1.053~1.489, and *P* = 0.011; dominant model: OR = 1.247, 95% CI = 1.039~1.497, and *P* = 0.018; UC: allele model: OR = 1.283, 95% CI = 1.085~1.518, and *P* = 0.004; dominant model: OR = 1.233, 95% CI = 1.030~1.475, and *P* = 0.023) (Figures 3(c) and 3(d)).

### 3.3. Meta-Analysis of the Association between* TLR4* Gene rs4986791C>T Polymorphism and IBD Risk

Nine studies investigated the association between* TLR4* rs4986791C>T polymorphism and IBD risk. No heterogeneity was observed in this meta-analysis (allele model: *I* ^2^ = 44.929%, *P*_*h*_ = 0.031; dominant gene model: *I* ^2^ = 33.948%, *P*_*h*_ = 0.097), and thus fixed effects model was adopted. The meta-analysis results showed that rs4986791C>T significantly increased IBD risk (allele model: OR = 1.259, 95% CI = 1.092~1.453, and *P* = 0.002; dominant gene model: OR = 1.246, 95% CI = 1.072~1.447, and *P* = 0.004) (Figures 2(c) and 2(d) and Table 3). Subgroup analysis based on ethnicity demonstrated that* TLR4* polymorphism rs4986791C>T was associated with an increased risk of IBD risk in both Asian and Caucasian populations (Asians: allele model: OR = 1.608, 95% CI = 1.080~2.395, and *P* = 0.019; dominant model: OR = 1.517, 95% CI = 1.000~2.303, and *P* = 0.050; Caucasians: allele model: OR = 1.215, 95% CI = 1.042~1.415, and *P* = 0.013; dominant model: OR = 1.210, 95% CI = 1.030~1.421, and *P* = 0.020) (Figures 3(e) and 3(f)). Interestingly, subgroup analysis based on the diseases type suggested that* TLR4* polymorphism rs4986791C>T conferred an increased risk of UC (allele model: OR = 1.304, 95% CI = 1.060~1.604, and *P* = 0.012; dominant model: OR = 1.276, 95% CI = 1.026~1.588, and *P* = 0.028), but CD did not exhibit such an association with* TLR4 *polymorphism rs4986791C>T (allele model: OR = 1.220, 95% CI = 0.998~1.486, and *P* = 0.058; dominant model: OR = 1.219, 95% CI = 0.991~1.499, and *P* = 0.061) (Figures 3(g) and 3(h)).

### 3.4. Potential Sources of Heterogeneity

**Figure 4:**
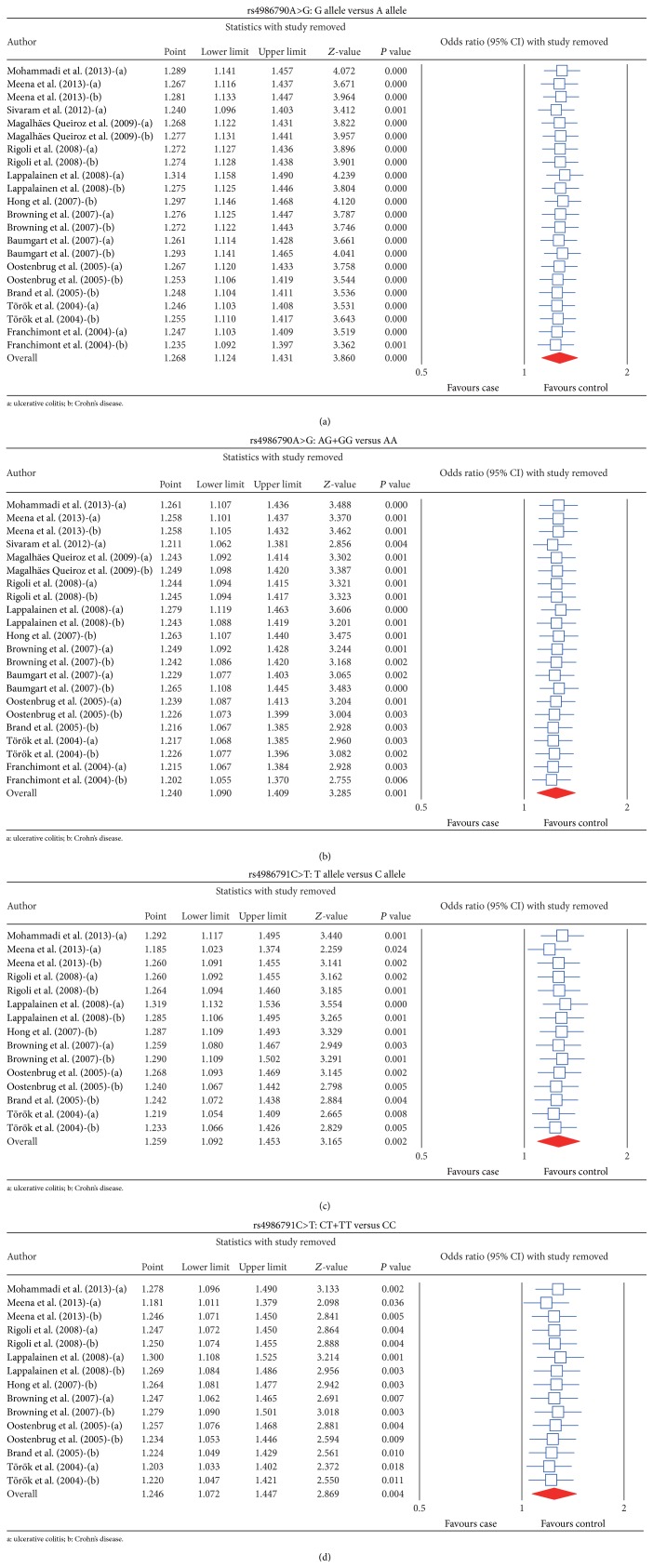
Sensitivity analysis of the differences in allele and genotype frequencies of TLR4 rs4986790A>G and rs4986791C>T polymorphism between case and control groups. (a) Sensitivity analysis of the differences in allele frequencies of rs4986790A>G; (b) sensitivity analysis of the differences in genotype frequencies of rs4986790A>G; (c) sensitivity analysis of the differences in allele frequencies of rs4986791C>T; (d) sensitivity analysis of the differences in genotype frequencies of rs4986791C>T.

**Figure 5:**
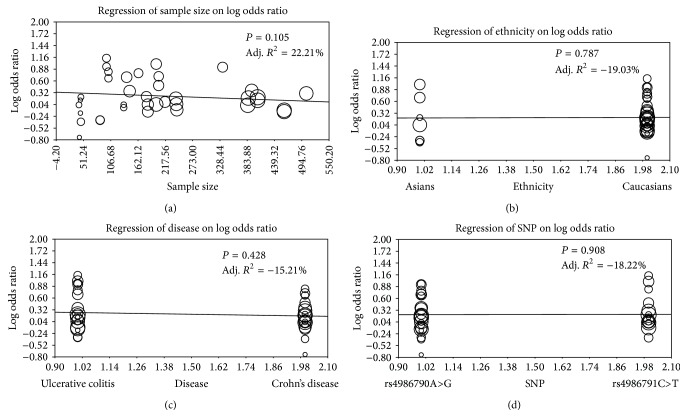
Metaregression analyses for potential source of heterogeneities on comparison of differences in allele and genotype frequencies of TLR4 rs4986790A>G and rs4986791C>T polymorphisms between case and control groups. (a) Metaregression analysis on sample size between case and control groups. (b) Metaregression analysis on ethnicity between case and control groups. (c) Metaregression analysis on disease between case and control groups. (d) Metaregression analysis on SNP between case and control groups.

**Figure 6:**
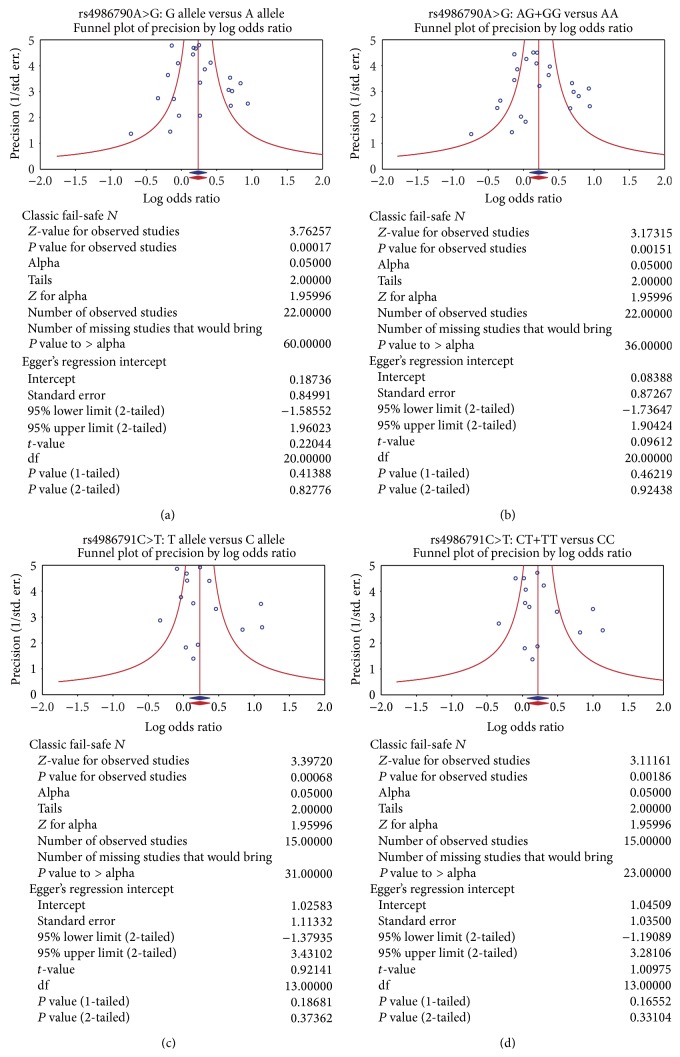
Publication bias of the differences in allele and genotype frequencies of TLR4 rs4986790A>G and rs4986791C>T polymorphism between case and control groups. (a) Publication bias of the differences in allele frequencies of rs4986790A>G; (b) publication bias of the differences in genotype frequencies of rs4986790A>G; (c) publication bias of the differences in allele frequencies of rs4986791C>T; (d) publication bias of the differences in genotype frequencies of rs4986791C>T.

Sensitivity analysis demonstrated that no single study had a significant effect on pooled ORs of the association of* TLR4 *rs4986790A>G and* TLR4 *rs4986791C>T polymorphisms with IBD susceptibility (Figure 4). The univariate meta-regression analysis showed that none of sample size, ethnicity, disease and SNP were main sources of heterogeneity and key factors for influencing overall effect size (all *P* > 0.05) (Figure 5). The multivariate metaregression analysis also identified that sample size, ethnicity, disease and SNP were not the potential sources of heterogeneity (all *P* > 0.05) (Table 4). The shape of the funnel plots did not reveal asymmetry and the statistical results did not indicate publication bias. Classic fail-safe *N* and Egger's linear regression test further confirmed the lack of significant publication bias (all *P* > 0.05) (Figure 6).

## 4. Discussion

In this study, the meta-analyses results showed that* TLR4 *polymorphisms rs4986790A>G and rs4986791C>T were associated with an increased susceptibility to IBD, implying that these SNPs are significant genetic risk factors for IBD. TLR4 is one of the key mediators of host immune responses towards bacteria and viruses [38]. Single nucleotide polymorphisms in* TLR4* can lead to abnormal signaling by altering the ligand binding of TLR4 and create an imbalance between pro- and anti-inflammatory cytokine secretion, resulting in the risk of chronic inflammation [39]. Functional* TLR4* gene polymorphisms create variations in the receptor domain responsible for recognition of pathogen-associated molecular pattern and, thereby, modulate immune response towards Th1 phenotype [28]. In addition, polymorphisms in other genes that are expressed in antigen-presenting cells, such as CD4, may also cause an inappropriate activation and polarization of T cells, as well as activating nuclear factor kappa B (NF-*κ*B), a key transcription factor related to inflammation [16].

TLR4 SNPs show unique distribution patterns in different populations from Africa, Asia, and Europe, and malarial infection is proposed as an explanation for these differences [40]. For example, Ioana et al. showed a mixed intermediate pattern of TLR4 SNPs in Iranian population compared to the commonly found patterns in the Americas, Europe, Africa, and Eastern Asia, which suggests the absence or weakness of selection pressure influencing TLR4 polymorphisms in this population [17]. In this context, two important SNPs in* TLR4* gene used in this study, rs4986790A>G and rs4986791C>T, alter the response of TLR4 receptor to LPS [12]. The interaction between LPS and TLR4 is a multistep process that LPS is present in circulation as a bound form with lipopolysaccharide-binding protein, which acts as an opsonin for CD14. CD14 subsequently catalyzes the binding of LPS to myeloid differentiation protein-2 (MD-2) and this allows LPS to be transferred to MD-2 and further facilitate a direct interaction with TLR4 to form a new LPS/MD-2/TLR4 complex [41]. TLR4 is expressed in macrophages, dendritic cells, and endothelial cells. Interestingly, intestinal epithelial cells only express TLR4 at low levels and this may explain why these cells are reasonably tolerant to LPS, possibly to prevent a full-blown immune response that could easily be triggered by the presence of a large number of colonizing bacteria in the intestinal lumen [30]. TLR4 mediates lipopolysaccharide-induced maturation and activation of myeloid dendritic cells and TLR4 upregulation in colonic mucosal epithelium and myeloid dendritic cells is observed in patients with active IBD [16]. The association of IBD with genetic variants in the* TLR4* gene has been previously reported but the results are contradictory, which may be explained by genetic heterogeneity between populations, stratification bias, or small sample size [15, 42]. Meena et al. reported that* TLR4* rs4986790A>G polymorphism is also associated with inflammatory bowel disease in North Indian population and further demonstrated its role in modulating the expression of inflammatory cytokines, leading to aberrant immune response in UC [28]. Collectively, these results discussed above suggest that* TLR4* rs4986790A>G and rs4986791C>T polymorphisms confer increased susceptibility to IBD.

It is clear from the discussion above that ethnic differences may potentially influence the impact of* TLR4* rs4986790A>G and rs4986791C>T polymorphisms on IBD progression. Subgroup analyses based on ethnicity and IBD disease type were performed to identify such differences. Our ethnicity-stratified analysis demonstrated that TLR4 rs4986790A>G and rs4986791C>T polymorphisms were associated with an elevated susceptibility to IBDs in both Asian and Caucasian populations. Subgroup analysis based on IBD disease type suggested that rs4986790A>G polymorphism increases the risk of both UC and CD. Interestingly, our findings showed that* TLR4* rs4986791C>T polymorphism may increase the susceptibility to UC but did not influence the risk of CD.

Limitations of our study should be noted while interpreting the result of our meta-analysis. First, our lack of access to the original data from the included studies limited a detailed assessment of potential interacting factors. Second, in the current meta-analysis the majority of the 13 eligible studies were performed in Caucasians and Asians, which may lead to bias. Moreover, studies published in languages other than English were also not included in our meta-analysis and, therefore, potentially relevant studies that could have influenced the results of this study may have been missed.

In conclusion,* TLR4* SNPs, rs4986790A>G and rs4986791C>T, are intimately associated with increased risk of IBD. Although the specific role of these polymorphisms in the etiopathogenesis of IBD and its two disease types remains elusive, it is possible that future studies may reveal the molecular mechanisms of IBD through experimental studies focused on these polymorphisms.

## Figures and Tables

**Table 1:** MAF of the SNPs in different ethnic groups from the HapMap population.

Ethnicity	rs4986790A>G	rs4986791C>T
CEU		
Alleles	A:G	C:T
MAF	0.033	0.033
CHB		
Alleles	A:A	C:C
MAF	0	0
YRI		
Alleles	A:G	C:C
MAF	0.033	0
JPT		
Alleles	A:A	C:C
MAF	0	0
JPT + CHB		
Alleles	A:A	C:C
MAF	0	0

CEU: 30 trios of Utah residents of Northern and Western European ancestry; CHB: 45 unrelated Han Chinese individuals from Beijing, China; YRI: 30 adult-and-both-parents Yoruba trios from Ibadan, Nigeria; JPT: 44 unrelated Japanese individuals from Tokyo, Japan; JPT + CHB: combined panel of Japanese in Tokyo, Japan, and Han Chinese in Beijing, China; MAF: minimum minor allele frequency; SNP: single nucleotide polymorphism.

**Table 2:** Baseline characteristics of included studies.

First author	Year	Country	Ethnicity	Total	Sample size		Gender (M/F)		Age (years)		Genotyping methods	SNP
					Case	Control	Case	Control	Case	Control		
Mohammadi^a^ [15]	2013	Iran	Asians	341	85	256	38/47	136/120	38 ± 16	37 ± 12	PCR-RFLP	S1 & S2
Meena^a^ [28]	2013	India	Asians	400	199	201	122/77	118/83	34.9 ± 11.6	36.4 ± 14.1	PCR-RFLP	S1 & S2
Meena^b^ [28]	2013	India	Asians	247	46	201	25/21	118/83	32.58 ± 11.05	36.4 ± 14.1	PCR-RFLP	S1 & S2
Sivaram^a^ [10]	2012	India	Asians	315	139	176	187/128		40.97 ± 14.11 (18–80)		AS-PCR	S1
Magalhäes Queiroz^a^ [29]	2009	Brazil	Caucasians	583	42	541	6/36	409/132	38.93 ± 14.73	33.87 ± 9.96	PCR-RFLP	S1
Magalhäes Queiroz^b^ [29]	2009	Brazil	Caucasians	584	43	541	20/23	409/132	40.88 ± 14.16	33.87 ± 9.96	PCR-RFLP	S1
Rigoli^a^ [30]	2008	Italy	Caucasians	148	45	103	27/18	68/35	43.2 ± 11.0	46.6 ± 9.8	PCR-RFLP	S1 & S2
Rigoli^b^ [30]	2008	Italy	Caucasians	236	133	103	70/63	68/35	43.5 ± 10.7	46.6 ± 9.8	PCR-RFLP	S1 & S2
Lappalainen^a^ [31]	2008	Finland	Caucasians	649	459	190	NR		NR		PCR-RFLP	S1 & S2
Lappalainen^b^ [31]	2008	Finland	Caucasians	430	240	190	NR		NR		PCR-RFLP	S1 & S2
Hong^b^ [32]	2007	New Zealand	Caucasians	370	182	188	NR		NR		PCR-RFLP	S1 & S2
Browning^a^ [33]	2007	New Zealand	Caucasians	821	405	416	53/214	240/176	NR		TaqMan assay	S1 & S2
Browning^b^ [33]	2007	New Zealand	Caucasians	805	389	416	64/250	240/176	NR		TaqMan assay	S1 & S2
Baumgart^a^ [16]	2007	Germany	Caucasians	548	145	403	67/78	NR	31 ± 13.6	NR	PCR-RFLP	S1
Baumgart^b^ [16]	2007	Germany	Caucasians	638	235	403	90/145	NR	26 ± 10.3	NR	PCR-RFLP	S1
Oostenbrug^a^ [34]	2005	Netherlands	Caucasians	516	217	299	NR		NR		PCR-RFLP	S1 & S2
Oostenbrug^b^ [34]	2005	Netherlands	Caucasians	803	504	299	NR		NR		PCR-RFLP	S1 & S2
Brand^b^ [35]	2005	Germany	Caucasians	403	204	199	96/108	99/100	37.8 ± 11.8	46.4 ± 15.3	AS-PCR	S1 & S2
Török^a^ [36]	2004	Germany	Caucasians	243	98	145	45/53	71/74	42.7 ± 13.3	44.6 ± 12.5	PCR-RFLP	S1 & S2
Török^b^ [36]	2004	Germany	Caucasians	247	102	145	37/65	71/74	40.9 ± 13.7	44.6 ± 12.5	PCR-RFLP	S1 & S2
Franchimont^a^ [37]	2004	Belgium	Caucasians	302	163	139	85/78	NR	29.78 ± 12.8	NR	PCR-RFLP	S1
Franchimont^b^ [37]	2004	Belgium	Caucasians	473	334	139	136/198	NR	26.6 ± 10.3	NR	PCR-RFLP	S1

M: male; F: female; SNP: single nucleotide polymorphism; ^a^ulcerative colitis; ^b^Crohn's disease; NR: not reported; PCR-RFLP: polymerase chain reaction-restriction fragment length polymorphism; AS-PCR: allele-specific polymerase chain reaction; S1: rs4986790A>G; S2: rs4986791C>T.

**Table 3:** Comparisons of genotype and allele frequencies between the case and the control groups.

Gene model		rs4986790A>G			rs4986791C>T		
		OR	95% CI	*P*	OR	95% CI	*P*
M allele versus W allele (allele model)	Overall	1.268	1.124~1.431	<0.001	1.259	1.092~1.453	0.002
	Ethnicity						
	Asians	1.246	0.945~1.642	0.119	1.608	1.080~2.395	0.019
	Caucasians	1.273	1.114~1.456	<0.001	1.215	1.042~1.415	0.013
	Disease						
	CD	1.242	1.053~1.489	0.011	1.220	0.998~1.486	0.058
	UC	1.283	1.085~1.518	0.004	1.304	1.060~1.604	0.012

WM + MM versus WW (dominant model)	Overall	1.240	1.090~1.409	0.001	1.246	1.072~1.447	0.004
	Ethnicity						
	Asians	1.099	0.812~1.489	0.540	1.517	1.000~2.303	0.050
	Caucasians	1.272	1.105~1.466	0.001	1.210	1.030~1.421	0.020
	Disease						
	CD	1.247	1.039~1.497	0.018	1.219	0.991~1.499	0.061
	UC	1.233	1.030~1.475	0.023	1.276	1.026~1.588	0.028

MM versus WW (homozygous model)	Overall	2.438	1.413~4.204	0.001	1.933	0.971~3.850	0.061

WM versus MM (heterozygous model)	Overall	0.477	0.272~0.836	0.010	0.625	0.308~1.268	0.193

MM versus WW + WM (recessive model)	Overall	3.211	1.840~5.606	<0.001	1.891	0.950~3.764	0.070

OR: odds ratio; 95% CI: 95% confidential intervals; UC: ulcerative colitis; CD: Crohn's disease.

**Table 4:** Metaregression analyses of potential source of heterogeneity.

Heterogeneity factors	Coefficient	SE	*t*	*P* (adjusted)	95% CI	
					LL	UL
Sample size	−0.001	0.001	−1.93	0.066	−0.001	0.001
Ethnicity	0.223	0.184	1.21	0.232	−0.152	0.598
Disease	−0.135	0.129	−1.05	0.325	−0.398	0.127
SNP	0.037	0.126	0.29	0.764	−0.219	0.293

SE: standard error; LL: lower limit; UL: upper limit; SNP: single nucleotide polymorphism.
